# Obstructive sleep apnoea and Alzheimer’s disease: In search of shared pathomechanisms

**DOI:** 10.1016/j.neubiorev.2017.12.004

**Published:** 2018-03

**Authors:** D. Polsek, N. Gildeh, D. Cash, R. Winsky-Sommerer, S.C.R. Williams, F. Turkheimer, G.D. Leschziner, M.J. Morrell, I. Rosenzweig

**Affiliations:** a Sleep and Brain Plasticity Centre, CNS, IoPPN, King’s College London, UK; b Croatian Institute for Brain Research, Medical School, University of Zagreb, Croatia; c Sleep Disorders Centre, Guy’s and St Thomas’ Hospital, London, UK; d Department of Neuroimaging, IoPPN, King’s College London, UK; e Surrey Sleep Research Centre, Department of Clinical and Experimental Medicine, Faculty of Health and Medical Sciences, University of Surrey, Guildford, UK; f Department of Neurology, Guy’s and St Thomas’ Hospital, London, UK; g Academic Unit of Sleep and Breathing, National Heart and Lung Institute, Imperial College London, UK and NIHR Respiratory Disease Biomedical Research Unit at the Royal Brompton and Harefield NHS Foundation Trust and Imperial College London, UK

**Keywords:** Obstructive sleep apnea, Alzheimer’s disease, Neuroinflammation, Astrocytes

## Abstract

Alzheimer’s disease (AD) is a significant public health concern. The incidence continues to rise, and it is set to be over one million in the UK by 2025. The processes involved in the pathogenesis of AD have been shown to overlap with those found in cognitive decline in patients with Obstructive Sleep Apnoea (OSA). Currently, the standard treatment for OSA is Continuous Positive Airway Pressure. Adherence to treatment can, however, be an issue, especially in patients with dementia. Also, not all patients respond adequately, necessitating the use of additional treatments. Based on the body of data, we here suggest that excessive and prolonged neuronal activity might contribute to genesis and acceleration of both AD and OSA in the absence of appropriately structured sleep. Further, we argue that external factors, including systemic inflammation and obesity, are likely to interfere with immunological processes of the brain, and further promote disease progression. If this hypothesis is proven in future studies, it could have far-reaching clinical translational implications, as well as implications for future treatment strategies in OSA.

## Highlights

• Alzheimer’s disease (AD) is a significant public health concern.
• The processes involved in the pathogenesis of AD are shown to overlap with those found in cognitive decline in Obstructive Sleep Apnoea (OSA).
• An excessive and prolonged neuronal activity might contribute to genesis and acceleration of both AD and OSA.
• External factors, such are systemic inflammation and obesity, can interfere with immunological processes of the brain, and promote disease progression.

## 1 Introduction

Alzheimer’s disease (AD) is one of the most significant public health challenges of the 21st century, affecting an ever increasing number of people. (Cedernaes et al., 2016, Mander et al., 2016) In the UK, the number of people with dementia is forecast to increase to over 1 million by 2025, and there are over 6 million people with dementia in Europe. (Association, 2016) In the United States, someone develops AD every 66 s. (Association, 2016) The AD worldwide epidemic has been touted as the only disease among the top 10 causes of death in the developed world that cannot at present be prevented, cured or even slowed. (Association, 2016) To date, despite significant scientific efforts, disappointingly little has been achieved in the way of effective prevention and therapeutic intervention for AD, and the urgency for divergent thinking on how to tackle this epidemic has been recognized. To that end, sleep has been proposed as a promising candidate that may serve as both a biomarker of AD, and as a new potentially therapeutic and preventative strategy for lowering AD risk. (Dissel et al., 2015, Ju et al., 2014, Kang et al., 2009, Mander et al., 2016)

In the community setting patients with mild to moderate AD frequently suffer with agitated behaviour at sunset. They and their carers also frequently report insomnia and fragmented sleep during the night, and excessive sleeping during the daytime, the intensity of which correlates with the severity of dementia. (Moran et al., 2005) Sleep disruption constitutes a core component of AD, and signature abnormalities of sleep have been shown to emerge well before clinical onset of AD. (Ju et al., 2014, Yaffe et al., 2014) Patients with amnestic mild cognitive impairment show EEG abnormalities during sleep, including fewer sleep spindles and reduced slow-wave sleep (SWS). (Ju et al., 2014, Westerberg et al., 2012) Similar sleep impairments are also present in older adults that are carriers of the apolipoprotein E (APOE) ε4 allele. The APOE ε4 allele is the most prominent genetic risk factor for late-onset AD, and, perhaps unsurprisingly, also one of the recognized risk factors for developing the second most common sleep disorder, obstructive sleep apnoea (OSA) (Mander et al., 2016). In OSA, sleep EEG signature abnormalities analogous to those encountered in AD have been independently reported (D’Rozario et al., 2016). Of note, thus far neither the time course of changes in the sleep EEG from preclinical to the clinical stages of dementia, nor its trajectory during the course of OSA process, have been fully documented or understood. (Ju et al., 2014)

**Fig. 1:**
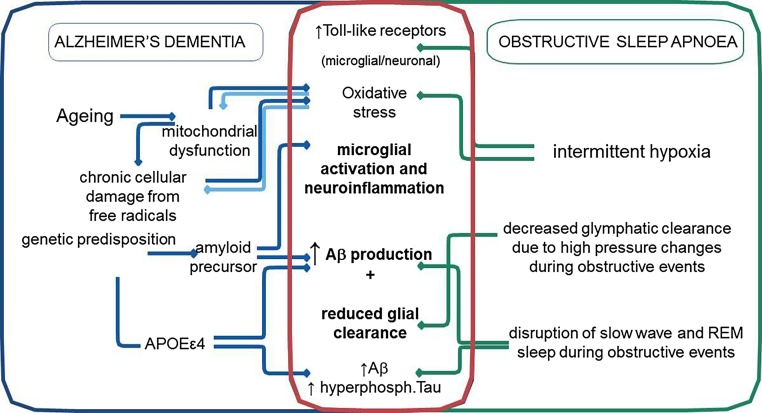
The schematic representation of proposed shared mechanisms between Alzheimer’s Disease (AD) and Obstructive Sleep Apnoea (OSA) (also refer to the text). *Abbreviations: SWS: slow wave sleep; Aβ:* amyloid-β peptide; APOE ε4: apolipoprotein E (APOE) ε4; TLR2: Toll-like receptor 2.

OSA has long been argued to share an epidemiological overlap (Fig. 1) and a bidirectional, causal relationship with AD (Cedernaes et al., 2016, Emamian et al., 2016, Pan and Kastin, 2014). OSA is a highly prevalent, debilitating chronic multi-system disorder, with strong links to obesity and older age (Lévy et al., 2015). Recent estimates suggest that up to 30% of men, and 12% of women, between 30 and 70 years of age are affected by OSA. (Peppard et al., 2013) It is characterized by brief periods of repetitive upper airway occlusion during sleep, leading to periods of intermittent hypoxia, hyper/hypocapnia, significant sleep fragmentation (Jordan et al., 2014), oxidative stress, and a chronic low-grade systemic inflammatory state. (Lévy et al., 2015) Some of the co-morbidities associated with untreated OSA include hypertension, stroke, diabetes, cardiac arrhythmias, myocardial infarction, heart failure, kidney disease, cancer and neurocognitive deficits and depression (Daulatzai, 2015, Gildeh et al., 2016, Lévy et al., 2015). It has been reported that individuals with OSA may convert to mild cognitive impairment (MCI) and AD at a younger age. (Osorio et al., 2015) Conversely, successfully treating OSA has been shown to delay the age of onset into mild cognitive impairment(Osorio et al., 2015) and to improve cognitive function in AD(Ancoli-Israel et al., 2008, Cooke et al., 2009). The results of recent *meta*-analysis suggest that patients with AD may have five times higher risk of presenting with OSA, compared to cognitively intact individuals of similar age. (Emamian et al., 2016) It has been further suggested that around 50% of patients with AD will have experienced OSA at some time after their initial diagnosis, negatively impacting on its prognosis and the quality of life. (Emamian et al., 2016)

The etiological roots of mechanisms behind effects of OSA on the central nervous system (CNS) have been extensively debated over the years (Gozal, 2013, Rosenzweig et al., 2013b), with some advocating the importance of disturbed sleep (Lim et al., 2013, Rosenzweig et al., 2014), and others championing the importance of oxidative stress and neuroinflammation over the former (Lavie, 2015, Rosenzweig et al., 2015, Yaffe et al., 2011). On balance, the consensus in the field is that an intricate interplay of all maladaptive and homeostatic adaptive processes instigated by OSA plays a part. Depending on the idiosyncratic physiological milieu and the severity, intensity and frequency of insults, this likely gives rise to OSA’s signature neurological deficits, as suggested by a number of neuroimaging and cognitive studies to date. (Kylstra et al., 2013, Rosenzweig et al., 2016, Rosenzweig et al., 2015, Tahmasian et al., 2016) In this opinion and hypotheses generating review, we build on some of these findings, and we use some of the most recent theories to extricate and propose several novel processes that might act as a shared mechanistic pathway between AD and OSA.

## 2 Sleep architecture or microstructure disturbances as a shared pathway in pathogenesis of OSA and AD

Signature changes in the sleep electroencephalographic (EEG) microstructure in OSA have been shown to include reduced slow wave activity and sigma power (reduced spindle activity) during non-rapid eye movement (NREM) sleep, along with slowing of the EEG during REM sleep (D’Rozario et al., 2016). At the very core of these changes are intercalated increased discrete events or bursts of increased neuronal activity, closely tied to periods of apneic breathing and associated EEG arousals, resulting in overall sleep deprivation and fragmentation of sleep. Sleep fragmentation in OSA has been associated with cognitive decline in a longitudinal study of patients (Cohen-Zion et al., 2004), and reported to be the most reliable predictor of episodic memory deficits in this patient group (Daurat et al., 2008). The seminal role of sleep in the regulation of CNS amyloid burden, and less conclusively in the regulation of tau levels in the brain, has recently been comprehensively argued (for further discussion of original studies refer to (Cedernaes et al., 2016, Ju et al., 2014, Mander et al., 2016).

The amyloid cascade hypothesis has been one of the most influential theoretical models of AD pathology. (Karran et al., 2011) The hypothesis posits that the imbalance between the production and clearance of amyloid-β (Aβ) peptide in the brain is the initiating and central event in AD pathology, ultimately leading to neurodegeneration and dementia. (Blennow et al., 2006, Karran et al., 2011). In the earliest stage of preclinical AD, soluble Aβ becomes insoluble and aggregates into amyloid plaques, leading to a reduction in soluble Aβ_42_ levels in the cerebrospinal fluid (CSF) (Blennow et al., 2006) Soluble Aβ in the interstitial fluid (ISF) has been shown to decrease during sleep and to increase during wakefulness. (Ju et al., 2016) Another hallmark of AD, tau pathology, has been shown to start early in the disease process in neurons in the medial temporal lobe, more specifically in the *trans*-entorhinal region, and to spread to the hippocampus and amygdala, and later to the neocortical association areas (Blennow et al., 2006). Significant correlations between subjective and objective measures of poor sleep with the severity of cortical Aβ burden, CSF measures of Aβ, and phosphorylated tau in CSF have been demonstrated in cognitively normal older adults, MCI and AD patients (Mander et al., 2016). In animals, the hypothalamic neuropeptide orexin-A has been implicated in this regulation, but the evidence from human studies has been less consistent (Cedernaes et al., 2016). Impaired function of orexinergic neurons has also been reported in patients with OSA, but the underlying mechanisms have not been fully clarified (Dergacheva et al., 2016). On the other hand, the orexinergic system was shown to be dysregulated in AD, where its output and function appeared to be overexpressed along the progression of the neurodegenerative process (Liguori et al., 2014). Additionally, disruption of circadian rhythms has been shown to precede the clinical onset of AD in some patients (Cedernaes et al., 2016). Similarly, in rodent models, experimentally induced sleep disruption has been shown to lead to several interlinked processes, otherwise independently implicated in development and progression of AD: an accumulation of Aβ and tau pathology, an increase in CNS oxidative stress, and reduction of the structural and functional integrity of the blood brain barrier (Cedernaes et al., 2016, Heneka et al., 2015). Of note, in a Drosophila model of AD-like pathology it has been shown that experimentally increasing sleep could restore long-term memory (Dissel et al., 2017). A recent study has shown that neuronally derived proteins are decreased in the CSF of patients with OSA compared to healthy controls (Ju et al., 2016). Liguori et al., 2017a, Liguori et al., 2017b also reported lower CSF Aβ42 concentrations, with higher CSF lactate levels, and higher t-tau/Aβ42 ratio compared to controls and treated patients. (Liguori et al., 2017b) It has been proposed that this could be due to impaired interaction between CSF and ISF in OSA (Ju et al., 2016). For instance, it is likely that during an obstructive apnea respiratory effort against a closed airway creates elevated intrathoracic and intracranial pressure along with a sudden pressure reversal at the end of the apnea, resulting in repetitive high-pressure fluctuations. These fluctuations may act to impede the glymphatic flow of metabolites from ISF into CSF leading to their retention and resulting in higher concentrations of Aβ, tau and other derived metabolites in the ISF (Ju et al., 2016). Moreover, in patients with OSA associated chronic hypertension, alongside cerebral amyloid angiopathy, can lead to increased stiffness of blood vessels that can further reduce clearance efficiency and accelerate amyloid accumulation in the CNS (Mander et al., 2016).

Whilst the exact role of particular sleep rhythms and stages in any aspect of AD pathology is uncertain, an increasing body of work supports the notion that NREM sleep may represent a pivotal pathway through which the brain manages Aβ levels. An interesting mechanistic model has emerged whereby slow wave activity (SWA) during NREM sleep has been described as the EEG indicator of reduced synaptic activity, with significantly decreased cortical metabolic rates (∼40%) compared to wakefulness. (Ju et al., 2016, Ju et al., 2014) Given that synaptic activity results in release of Aβ from neurons, it has been postulated that SWA leads to decreased production and decreased regional levels of Aβ (Ju et al., 2016, Mander et al., 2016). The amplitude of diurnal variation in Aβ concentration in healthy young adults has been estimated to be rather high, approximately around 30% peak-to-peak, suggesting that sleep patterns could considerably affect levels of soluble Aβ in ISF in states of chronic sleep disruption, such as OSA or behaviourally restricted sleep in individuals(Ju et al., 2014). Mander and colleagues (2016) have also long reasoned that disrupted NREM SWS and excess wakefulness increase Aβ aggregation, which they have argued itself impairs NREM SWS, resulting in a vicious cycle accelerating AD progression (Mander et al., 2016). Moreover, this group has also proposed the signature association between the low-frequency range of NREM SWA below 1 Hz (i.e., slow oscillations) with Aβ accumulation in the medial prefrontal cortex, one of the earliest sites known to accumulate Aβ in AD pathology. (Mander et al., 2016) In addition, another hallmark of AD, early neurofibrillary tangle (NFT) aggregation in the medial temporal lobe during AD progression has been linked to relative desynchronization of hippocampal ripples in rodents, and abnormally long hyperpolarized down-states and impaired depolarizing up-states during NREM slow oscillations (Mander et al., 2016). The associations between CSF tau and diminished NREM SWS have also been reported in clinical studies (Mander et al., 2016). Perhaps correspondingly, it has been shown that AD patients have fewer NREM sleep spindles relative to healthy older adults, with the degree of spindle reduction predicting the severity of memory impairment (Ancoli-Israel et al., 2008). In the same vein, in OSA patients, altered spindle activity has also been argued to indicate frontal thalamo-cortical dysfunction (Carvalho et al., 2014). The changes in the microstructure of NREM sleep have also been implicated, and their role investigated in cognitive impairment (Ferini-Strambi et al., 2013, Maestri et al., 2015). For example, cyclic alternating pattern (CAP), a spontaneous and physiological rhythm of NREM sleep, characterized by electroencephalographic oscillations believed to correspond to a condition of sustained arousal instability (Maestri et al., 2015). CAP rate, and CAP slow components (A1 index) were reported as decreased in MCI subjects, and to a greater extent in AD patients, compared to cognitively intact controls(Maestri et al., 2015).

Although the relationship between NREM disruption and AD pathology is recognised, this link has also been argued to extend to REM sleep disruption (Mander et al., 2016). One of the proposed functions of REM sleep is the regulation of emotional reactivity and mood states, both of which have been shown to be disturbed in AD patients (Mander et al., 2016). AD patients suffer with impaired enhancing effects of emotion on memory retention, and they express deficits in processing of complex emotional information (Mander et al., 2016). Furthermore, major depressive disorder and post-traumatic stress disorder, known to be linked with OSA, and associated with REM sleep disturbance, have been both recognized as risk factors for developing of AD (Mander et al., 2016, Rosenzweig et al., 2015). Additionally, reduced REM sleep amount, delayed REM sleep onset, and blunted rebound of REM sleep following selective deprivation have all been demonstrated in patients with MCI and AD (Mander et al., 2016). Moreover, reductions in the EEG quality of REM sleep have been proposed as a possible biomarker that could help discriminate those with AD from cognitively normal older adults (Hassainia et al., 1997) and REM sleep was recently shown to be a risk factor to develop dementia(Pase et al., 2017). The selective degeneration of cholinergic projection neurons within the brainstem and basal forebrain in AD brains, has been proposed as possible mechanism that may underlie this disruption (Blennow et al., 2006, Mander et al., 2016). In keeping, the degree of cortical Aβ burden has been shown to correlate with the degree of basal forebrain atrophy, due to amyloid angiopathy, in healthy older adults, MCI, and AD patients (Kerbler et al., 2015). Also, Aβ and tau burden have been both implicated in the degeneration of cholinergic neurons projecting from the basal forebrain to the cortex (Mander et al., 2016). Analogously, in a recent exploratory transcranial magnetic stimulation (TMS) study, impaired cognitive performance in OSA patients have been linked with a dysfunction of the cholinergic system (Nardone et al., 2016).

Whilst the discussed reciprocal mechanisms behind AD pathology and sleep disruption might not all be equally implicated in different phenotypes, or indeed stages of OSA, they nonetheless raise some valid questions and evidently argue novel treatment targets (Castronovo et al., 2014, Liguori et al., 2017a). After all, the current gold standard treatment for OSA, i.e. continuous positive airway pressure device (CPAP), is poorly tolerated by many patients, and the limited improvement and refractoriness of neurological deficits to CPAP treatment have been long recognised (Castronovo et al., 2014, Rosenzweig et al., 2015). In the future, depending on the sub-phenotype of OSA and its symptom constellation a personalized medical approach could warrant that alternative treatments and interventions in patients with OSA are used in combination with CPAP or other interventions and lifestyle factors. For instance, in dysphoric and anxious OSA patients with predominant cognitive problems, cholinesterase inhibitors might be prescribed. Cholinesterase inhibitors have been shown to increase REM sleep quality and duration (Mander et al., 2016), and they might arguably help with memory, mood and emotional symptoms in AD and OSA patients. Conversely, therapeutic interventions that have been shown to restore NREM SWS (e.g. auditory closed-loop stimulation (Ngo et al., 2013) or transcranial current stimulation) might be used as a preventative measure to reduce AD risk in younger patients, or in high-vulnerability populations, such as patients with Down’s syndrome or individuals carrying the ApoE ε4 allele with marked sleep deficits(Mander et al., 2016).

## 3 Neurogenic neuroinflammation as a shared pathway in pathogenesis of OSA and AD

Remarkably, taken together, the body of evidence appears to strongly argue for prolonged wakefulness, or rather the excessive neuronal synaptic activity, as “*unus mundus*”, the most unifying feature underlying the link between sleep and AD pathology. Aside from the sophisticated complexities of sleep-related effects, the evidence would suggest that it is the excessive neuronal synaptic activity itself that is sufficient to set in motion the maladaptive positive-feedback-forward loop through increased production of Aβ, higher neurometabolic rate and oxidative stress, which can then further interfere with sleep processes, and ultimately lead to accelerated AD pathophysiological progression(Mander et al., 2016). Some indirect support for this notion can be drawn from neuroimaging studies, where increased Aβ deposition has been shown to occur preferentially in multimodal brain regions corresponding to a default mode network and including the posterior cingulate cortex, parahippocampal gyrus and medial frontal cortex (Jagust and Mormino, 2011). These regions show continuous levels of heightened activation and plasticity across the lifespan, which could underlie their vulnerability (Jagust and Mormino, 2011, Ju et al., 2014). Moreover, neuroimaging studies of subjects with genetic predispositions to AD have also been consistent with these findings, suggesting a mechanism whereby neural efficiency or cognitive reserve may diminish Aβ deposition (Jagust and Mormino, 2011). In further support, it has been recently proposed that inflammatory responses in the CNS, including orchestrated actions of immune cells, vascular cells and neurons can be triggered by the increased neuronal activity. The technical term ‘neurogenic neuroinflammation' has been suggested (refer to (Xanthos and Sandkuhler, 2014).

### 3.1 Neurogenic neuroinflammation and the multipartite synapse

In comparison to the inflammatory responses to various insults that are readily induced in regenerating peripheral tissues, the relatively mild inflammatory tissue reactions in the CNS reflect lower regenerative capacity of neurons (Xanthos and Sandkuhler, 2014). It has been proposed that under normal conditions neurogenic neuroinflammation acts to maintain homeostasis and enables the CNS to cope with enhanced metabolic demands (Xanthos and Sandkuhler, 2014). It has been suggested that it may also modulate the computational power and plasticity in neuronal networks (Xanthos and Sandkuhler, 2014). This concept is not new and the seminal body of work by Attwell and colleagues, and other groups, supports the crucial role for multidirectional synapse cross-talks between astrocytes, neurons, glia and vascular smooth cells as powerful regulators of neuronal spiking, synaptic plasticity and brain blood flow (Bazargani and Attwell, 2016). Hence, it comes as no surprise that neuroinflammation has also been increasingly argued as a principal treatment target in people with AD (Heneka et al., 2015), and more recently in OSA (Daulatzai, 2015, Lavie, 2015). This has been further supported by the results of genome-wide analyses, which have shown that several genes that increase the risk for sporadic AD encode factors that regulate glial clearance of misfolded proteins and the inflammatory reaction (Heneka et al., 2015). Ageing brains of OSA patients in particular may provide a milieu for a chronic longitudinal priming of microglia, the resident mononuclear phagocytes, to various activators, such as chronic vascular changes, including cerebrovascular dysregulation and cerebral microinfarcts, local ischaemia, chronic exposure to Aβ, neuronal debris, at times of increasing imbalance between pro-oxidant and anti-oxidant physiological systems of the body(Lavie, 2015). Various exogenous and endogenous factors have also been speculated to further modify the innate immune response induced by Aβ-exposed microglia (Heneka et al., 2015). Apart from disturbed sleep, amongst other environmentally modifiable risk factors, systemic inflammation and obesity have been shown to affect risk through an increase in sustained neuroinflammatory drive (for further discussion and list of original studies refer to (Heneka et al., 2015)). Notably, co-morbid obesity has been linked in patients to increased propensity to acquire infections leading to systemic infection. (Heneka et al., 2015) Obesity-associated reduced gut microbial diversity has been associated with increased concentrations of proinflammatory markers in peripheral blood (Heneka et al., 2015). Also, white adipocyte tissue itself has been shown to have a high percentage of activated macrophages, a rich source of proinflammatory cytokines. (Heneka et al., 2015) Moreover, in a rodent model, obesity-associated type 2 diabetes has been reported to accelerate memory dysfunction and neuroinflammation. (Heneka et al., 2015)

Microglia and astrocytes have been shown to release cytokines, interleukins, nitric oxide (NO), and other potentially cytotoxic molecules after exposure to Aβ, thereby exacerbating the neuroinflammatory response (Heneka et al., 2015). Logic would dictate that part of any homeostatic drive during inflammatory processes would have to include increased clearance of byproducts of this sustained high neurometabolic rate. Sleep’s role in synaptic homeostasis (Cirelli and Tononi, 2015) has long been argued for, and a body of evidence also suggest that the clearance of neurometabolites predominantly occurs during sleep (Ju et al., 2014). The clearance of Aβ has been shown to depend on local degradation by a wide range of proteases, phagocytosis by glial cells, egress across the BBB, reabsorption through the CSF(Cedernaes et al., 2016), and most recently it has shown to occur via glymphatic system during sleep(Xie et al., 2013). Dysregulated microglia has also been recently shown to contribute to spread of tau pathology via synaptic and non-synaptic transmission, including via exocytosing microvesicles such as exosomes (Asai et al., 2015).

### 3.2 Multipartite synapse at the core of pathological changes in AD and OSA and its role in clearance of Aβ and tau pathology

**Fig. 2:**
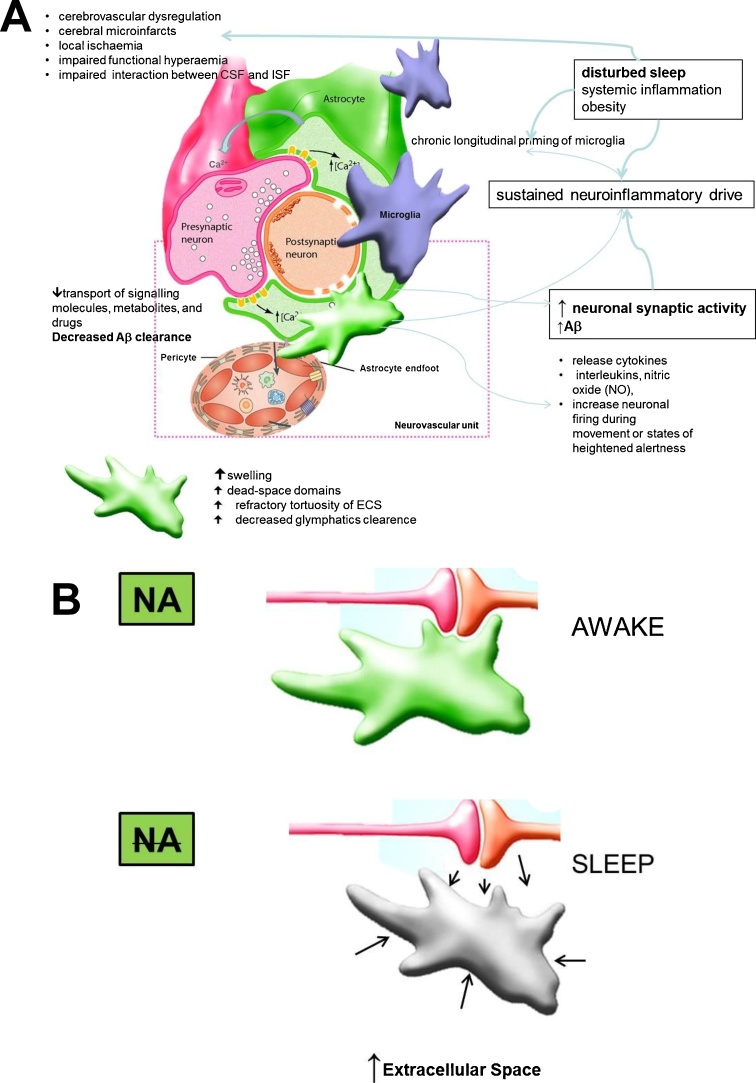
Proposed effects of disturbed sleep and altered neuronal activity at the level of multipartite synapse (A) (adapted from(Fellin et al., 2006, Xanthos and Sandkuhler, 2014). Increased clearance of metabolites has been postulated to occur during sleep, due to a low noradrenergic tone and decrease in astrocyte volume, resulting in increased extracellular space and increased glymphatic flow(B). (for in depth explanation refer to the main text). *Abbreviations: NA: noradrenaline; ECS: extracellular space; Aβ:* amyloid-β peptide*; CSF: cerebrospinal fluid; ISF; interstitial fluid.*

AD has been associated with distinct inflammatory, functional, and morphological multipartite synapse alterations including regional changes in cerebral blood vessels and perivascular glia and neurons (the neurovascular unit) (see Fig. 2A) (Heneka et al., 2015). These early-onset and progressive changes, which are induced by combined effects of soluble Aβ oligomers and vascular Aβ deposits, ultimately lead to decreased cerebral blood flow and impaired functional hyperaemia (ie, the ability of local blood flow to increase in response to neuronal activation). From a clinical perspective it is hence important to note that pharmacologically modulating inflammatory signalling pathways systemically or regionally (but not cell specifically) may inadvertently result in complex synergistic and/or antagonist interactions with unpredictable overall results(Xanthos and Sandkuhler, 2014). For instance, it is possible that previously reported slowing of wakeful EEG in both AD and OSA patient groups (D’Rozario et al., 2016) is not solely an epiphenomenon of underlying CNS changes, but that they in a fact present as a result of a still poorly understood homeostatic mechanism, which acts to decrease excessive neuronal and neurometabolic activity over the periods of wake to counteract insufficient periods of senescence during sleep (Ju et al., 2014, D’Rozario et al., 2016). Arguably this would not be that dissimilar to accepted exogenous iatrogenic intervention of placing a patient with an ongoing refractory status epilepticus under the general anesthesia. It perhaps follows, that at least theoretically, any forced non-personalised intervention, such as may occur during indiscriminately used CPAP intervention, might at least initially counteract adaptive aspects of inflammation before resolving the cause of the inflammatory process. This concept is yet to be confirmed in future prospective studies, but it may as such significantly change our approach to sleep management in OSA patients.

Another potentially interesting treatment target for AD would have to involve the regulation of the CNS extracellular space (Fig. 2B). The extracellular space (ECS) is possibly best described as an interconnected channel mesh that allows diffusion-mediated transport of signalling molecules, metabolites, and drugs (Sherpa et al., 2016). Astrocytes and their morphology have been increasingly implicated in regulation of this space under normal and pathological conditions. For instance, astrocytic swelling under conditions of ischaemia or inflammation increases so called the ‘dead-space domains’ in the ECS, which have been demonstrated even after recovery of the acute swelling(Sherpa et al., 2014). The possible clinical implication for AD and OSA patients would be that inflammation driven repeated hypoxic or hypotonic stress may with time lead to increasing refractory tortuosity of the ECS whereby toxic metabolites such as Aβ oligomers are trapped. Relatedly, it has been shown that the ECS decreases in part through an increase in astrocytic volume following β2 adrenergic receptor (β2AR) activation. This is of relevance as it implies close noradrenergic regulation of synaptic availability and extracellular concentration of neurotransmitters and neuromodulators that can help facilitate neuronal interactions, especially during wakefulness (Sherpa et al., 2016). Conversely, astrocytes have also been proposed to play an important role in clearance of soluble Aβ from the parenchyma by paravascular drainage, the recently described glymphatic system (Jessen et al., 2015). It has been suggested that this pathway depends on the astrocytic water channel aquaporin 4 (Jessen et al., 2015, Mander et al., 2016), although this notion was recently challenged(Smith et al., 2017). Whilst its function in human metabolic clearance is yet to be comprehensively demonstrated, indirect support has been provided by several recent neuroimaging studies (Bernardi et al., 2016, Cedernaes et al., 2016). In rodents, a 60% increase in the ECS has been shown during sleep (Xie et al., 2013), as compared with the space found during wakefulness, and sleep was found to increase the convective flow of ISF from the para-arterial to the para-venous space resulting in a doubling of the rate of Aβ removal (Cedernaes et al., 2016, Mander et al., 2016). Moreover, these effects were furthermore mimicked by infusion of noradrenergic receptor antagonists, suggesting that low adrenergic input is required for this convective clearance to occur (Xie et al., 2013).

Based on these findings it has been proposed that locus coeruleus (LC) quiescence during sleep may act as the main driver of metabolite clearance by lowering the adrenergic tone (Mander et al., 2016). LC, located at the dorsal part of the brain stem, is the main source of noradrenaline (NA) in the brain. The LC neurons project throughout the brain, where NA is released via axonal varicosities via volume transmission. The reciprocal monosynaptic pathways between the mPFC and LC have been previously demonstrated, and complex excitatory and inhibitory effects on cortical and subcortical cells, depending on concentration of the NA and on receptor distribution and affinity in the target region have been described (Atzori et al., 2016). Of relevance to AD pathology, in addition to its role as a neurotransmitter, NA has potent anti-inflammatory, anti-oxidative, neurotrophic, and neuroprotective actions. (Heneka et al., 2015) and LC-NA release has been postulated as a powerful central regulator of CNS spatio-temporal activation and energy expenditure (refer to (Atzori et al., 2016)). The number of cells in the LC, and concentration of NA in the brain, decrease during normal ageing, although more pronounced cell loss has been demonstrated in patients with AD. (Heneka et al., 2015) Thus, early degeneration of the LC and subsequent loss of NA-mediated innervation could substantially promote the inflammatory response to any stimulus, including Aβ. Similar degeneration has been observed in animal models of OSA (Zhu et al., 2007). However in patients with OSA due to technical limitations of brain stem and sleep imaging (Otte et al., 2016), no conclusive data are yet available. Experimental loss of NA has been shown to compromise microglial migration and Aβ phagocytosis *in vivo*, suggesting that a loss of NA tone increases not only inflammation, but also Aβ deposition (Heneka et al., 2015). We suggest that the importance of LC firing in AD and OSA pathology is hence twofold: its quiescence and low NA tone have been argued crucial for effective sleep-driven glymphatic clearance of metabolites, whilst its activity during wakefulness might regulate the ECS space, contribute to CNS spatio-temporal activation and consciousness states, and regulate energy expenditure (Atzori et al., 2016, Sherpa et al., 2014). Of note LC-NA system firing has been shown during NREM EEG slow oscillations, correlating with Down-to-Up state transition (Eschenko et al., 2012). Arguably, LC firing at the times of the cortico-hippocampal neuronal replay might suggest a novel and intriguing role for the LC in sleep-dependent memory consolidation (Eschenko et al., 2012). This finding might also be of particular importance for OSA pathology, as any such arousal-driven activation of LC during the night might inappropriately hijack its role with serious consequences for sleep-mediated memory consolidation and plasticity (Rosenzweig et al., 2016, Twigg et al., 2010). Moreover, on a more mechanistic level, LC-NA system firing during NREM EEG slow oscillations might also have a pure mechanistic pulsatile effect through astrocytes-driven changes in ECS, providing a more efficient clearance at times of synchronised neuronal activity (Fig. 2). It would follow, that in OSA and AD, any such mechanism would also be misappropriated and impaired with serious consequences. Future electrophysiological and neuroimaging studies should help elucidate these intriguing theoretical possibilities.

Finally, the functional connection of AD and OSA with endogenous neurogenesis remains a lingering question in the field. Neurogenesis – the formation of new neurons in the adult brain – is considered to be one of the mechanisms by which the brain maintains its lifelong plasticity in response to extrinsic and intrinsic changes. In OSA hippocampal hypertrophy in a cohort of patients with predominantly mild OSA has been demonstrated, whereby it has been argued that altered endogenous neuroglia genesis might play a part in the homeostatic adaptive process to a mild injury in some patients. (Rosenzweig et al., 2013a) One of the new promising therapeutic avenues for treatment of AD pathology might be via enhancing endogenous neurogenesis and promotion of compensatory role for newborn neurons in dementia. In keeping, findings from number of animal and clinical studies, mainly from the previous non-English speaking (e.g. previous USSR) countries, have suggested that intermittent hypoxia might have bidirectional relationship with endogenous neurogenesis as a part of the adaptive homeostatic ischaemic pre/postconditioning processes (Mateika and Komnenov, 2017). Perhaps relatedly, Ekonomou and colleagues (2015) recently reported that severe AD pathology impaired the production of new neurons, and that there was a significant positive correlation between the cell numbers of activated microglia and those of the newly generated neurons (Ekonomou et al., 2015). It has been previously proposed that microglia sense signals from the surrounding environment and have regulatory effects, both proneurogenic and antineurogenic, on adult neurogenesis (Heneka et al., 2015, Rolls et al., 2007). In support, microglial Toll-like receptor 2 (TLR2) deficiency in mice has been shown to impair hippocampal neurogenesis(Rolls et al., 2007). TLR2 expression and activity has also been shown increased on monocytes of patients with OSA. (Chen et al., 2015) It arguably follows that any potential future AD or OSA neurogenic therapy would likely need to target neuroinflammation simultaneously to be effective.

## 4 Conclusion and future directions

The increasing body of data suggests that excessive and prolonged neuronal activity in the absence of appropriately structured sleep and periods of neuronal quiescence might contribute to genesis and acceleration of the neurodegenerative process in patients with AD. If this hypothesis is borne out in future studies, it could have far-reaching clinical translational implications, as well as implications for future treatment strategies in OSA. Currently, the standard treatment for OSA is CPAP treatment, which has been shown effective in slowing the cognitive decline in comorbid OSA and AD (Troussiere et al., 2014). However, the adherence to treatment can be an issue, especially in patients with dementia, and not all patients respond adequately, necessitating the use of additional treatments. In addition, external factors, including systemic inflammation and obesity, are likely to interfere with immunological processes of the brain and further promote disease progression. Modulation of risk factors and targeting of these immune mechanisms could lead to future therapeutic or preventive strategies for both co-morbidities, OSA and AD. For example, in certain individuals, by treating the neuroinflammation early (e.g. via anti-inflammatory agents) along with the CPAP treatment, even prior to the development of any neurocognitive pathology, the genesis, or slowing of subsequent pathology and later dementia might be possible. In addition, the possible role for early adaptive microglial activation to intermittent hypoxia in activation and promotion of endogenous neurogenesis could be an attractive therapeutic target. Similarly, by targeting pronounced sleep fragmentation and enhancing SWA (e.g. by non-invasive stimulation), Aβ production and clearance might be influenced.

## Conflict of interest statement

The authors declare that the research was conducted in the absence of any commercial or financial relationships that could be construed as a potential conflict of interest.

This work is in memory of a great teacher Andrew Huxley (1917–2012).

## References

[bib0005] Ancoli-Israel S., Palmer B.W., Cooke J.R., Corey-Bloom J., Fiorentino L., Natarajan L., Liu L., Ayalon L., He F., Loredo J.S. (2008). Cognitive effects of treating obstructive sleep apnea in Alzheimer's disease: a randomized controlled study. J. Am. Geriatr. Soc..

[bib0010] Asai H., Ikezu S., Tsunoda S., Medalla M., Luebke J., Haydar T., Wolozin B., Butovsky O., Kügler S., Ikezu T. (2015). lluding via exocytosing microvesicles such as exosomes. Nat. Neurosci..

[bib0015] Association (2016). Association A.s., 2016. 2016 ALZHEIMER'S DISEASE FACTS AND FIGURES.

[bib0020] Atzori M., Cuevas-Olguin R., Esquivel-Rendon E., Garcia-Oscos F., Salgado-Delgado R.C., Saderi N., Miranda-Morales M., Trevino M., Pineda J.C., Salgado H. (2016). Locus ceruleus norepinephrine release: a central regulator of CNS spatio-temporal activation?. Front. Synaptic. Neurosci..

[bib0025] Bernardi G., Cecchetti L., Siclari F., Buchmann A., Yu X., Handjaras G., Bellesi M., Ricciardi E., Kecskemeti S.R., Riedner B.A., Alexander A.L., Benca R.M., Ghilardi M.F., Pietrini P., Cirelli C., Tononi G. (2016). Sleep reverts changes in human gray and white matter caused by wake-dependent training. Neuroimage.

[bib0030] Blennow K., de Leon M.J., Zetterberg H. (2006). Alzheimer’'s disease. Lancet.

[bib0035] Carvalho D.Z., Gerhardt G.J., Dellagustin G., de Santa-Helena E.L., Lemke N., Segal A.Z., Schonwald S.V. (2014). Loss of sleep spindle frequency deceleration in obstructive sleep apnea. Clin. Neurophysiol..

[bib0040] Castronovo V., Scifo P., Castellano A., Aloia M.S., Iadanza A., Marelli S., Cappa S.F., Strambi L.F., Falini A. (2014). White matter integrity in obstructive sleep apnea before and after treatment. Sleep.

[bib0045] Cedernaes J., Osorio R.S., Varga A.W., Kam K., Schioth H.B., Benedict C. (2016). Candidate mechanisms underlying the association between sleep-wake disruptions and Alzheimer’s disease. Sleep Med. Rev..

[bib0050] Chen Y.C., Su M.C., Liou C.W., Liu S.F., Chen C.J., Lin H.C., Hsiao C.C., Wang T.Y., Wang C.C., Chin C.H., Huang K.T., Lin A.S., Lin M.C. (2015). Co-upregulation of toll-like receptors 2 and 6 on peripheral blood cells in patients with obstructive sleep apnea. Sleep Breath..

[bib0055] Cirelli C., Tononi G. (2015). Sleep and synaptic homeostasis. Sleep.

[bib0060] Cohen-Zion M., Stepnowsky C., Johnson S., Marler M., Dimsdale J.E., Ancoli-Israel S. (2004). Cognitive changes and sleep disordered breathing in elderly: differences in race. J. Psychosom. Res..

[bib0065] Cooke J.R., Ayalon L., Palmer B.W., Loredo J.S., Corey-Bloom J., Natarajan L., Liu L., Ancoli-Israel S. (2009). Sustained use of CPAP slows deterioration of cognition, sleep, and mood in patients with Alzheimer's disease and obstructive sleep apnea: a preliminary study. J. Clin. Sleep Med..

[bib0070] D’Rozario A.L., Cross N.E., Vakulin A., Bartlett D.J., Wong K.K.H., Wang D., Grunstein R.R. (2016). Quantitative electroencephalogram measures in adult obstructive sleep apnea – potential biomarkers of neurobehavioural functioning. Sleep Med. Rev..

[bib0075] Daulatzai M.A. (2015). Evidence of neurodegeneration in obstructive sleep apnea: relationship between obstructive sleep apnea and cognitive dysfunction in the elderly. J. Neurosci. Res..

[bib0080] Daurat A., Foret J., Bret-Dibat J.L., Fureix C., Tiberge M. (2008). Spatial and temporal memories are affected by sleep fragmentation in obstructive sleep apnea syndrome. J. Clin. Exp. Neuropsychol..

[bib0085] Dergacheva O., Yamanaka A., Schwartz A.R., Polotsky V.Y., Mendelowitz D. (2016). Hypoxia and hypercapnia inhibit hypothalamic orexin neurons in rats. J. Neurophysiol..

[bib0090] Dissel S., Angadi V., Kirszenblat L., Suzuki Y., Donlea J., Klose M., Koch Z., English D., Winsky-Sommerer R., van Swinderen B., Shaw P.J. (2015). Sleep restores behavioral plasticity to Drosophila mutants. Curr. Biol..

[bib0095] Dissel S.K.M., Donlea J., Cao L., English D., Winsky-Sommerer R., van Swinderen B., Shaw P.J. (2017). Sleep can be used as a therapeutic to reverse memory impairment and the underlying pathology in Drosophila models of Alzheimer’s disease. Neurobiol. Sleep Circadian Rhythms.

[bib0100] Ekonomou A., Savva G.M., Brayne C., Forster G., Francis P.T., Johnson M., Perry E.K., Attems J., Somani A., Minger S.L., Ballard C.G., Cognitive Medical Research Council Cognitive F F., Ageing Neuropathology S. (2015). Stage-specific changes in neurogenic and glial markers in Alzheimer’s disease. Biol. Psychiatry.

[bib0105] Emamian F., Khazaie H., Tahmasian M., Leschziner G.D., Morrell M.J., Hsiung G.Y., Rosenzweig I., Sepehry A.A. (2016). The association between obstructive sleep apnea and Alzheimer’s disease: a meta-analysis perspective. Front. Aging Neurosci..

[bib0110] Eschenko O., Magri C., Panzeri S., Sara S.J. (2012). Noradrenergic neurons of the locus coeruleus are phase locked to cortical up-down states during sleep. Cereb. Cortex.

[bib0115] Fellin T., Pascual O., Haydon P.G. (2006). Astrocytes coordinate synaptic networks: balanced excitation and inhibition. Physiology (Bethesda).

[bib0120] Ferini-Strambi L., Galbiati A., Marelli S. (2013). Sleep microstructure and memory function. Front. Neurol..

[bib0125] Gildeh N., Drakatos P., Higgins S., Rosenzweig I., Kent B.D. (2016). Emerging co-morbidities of obstructive sleep apnea: cognition, kidney disease, and cancer. J. Thorac. Dis..

[bib0130] Gozal D. (2013). CrossTalk proposal: the intermittent hypoxia attending severe obstructive sleep apnoea does lead to alterations in brain structure and function. J. Physiol..

[bib0135] Hassainia F., Petit D., Nielsen T., Gauthier S., Montplaisir J. (1997). Quantitative EEG and statistical mapping of wakefulness and REM sleep in the evaluation of mild to moderate Alzheimer’s disease. Eur. Neurol..

[bib0140] Heneka M.T., Carson M.J., El Khoury J., Landreth G.E., Brosseron F., Feinstein D.L., Jacobs A.H., Wyss-Coray T., Vitorica J., Ransohoff R.M., Herrup K., Frautschy S.A., Finsen B., Brown G.C., Verkhratsky A., Yamanaka K., Koistinaho J., Latz E., Halle A., Petzold G.C., Town T., Morgan D., Shinohara M.L., Perry V.H., Holmes C., Bazan N.G., Brooks D.J., Hunot S., Joseph B., Deigendesch N., Garaschuk O., Boddeke E., Dinarello C.A., Breitner J.C., Cole G.M., Golenbock D.T., Kummer M.P. (2015). Neuroinflammation in Alzheimer’s disease. Lancet Neurol..

[bib0145] Jagust W.J., Mormino E.C. (2011). Lifespan brain activity, beta-amyloid, and Alzheimer’s disease. Trends Cogn. Sci..

[bib0150] Jessen N.A., Munk A.S., Lundgaard I., Nedergaard M. (2015). The glymphatic system: a beginner’s guide. Neurochem. Res..

[bib0155] Jordan A.S., McSharry D.G., Malhotra A. (2014). Adult obstructive sleep apnoea. Lancet.

[bib0160] Ju Y.E., Lucey B.P., Holtzman D.M. (2014). Sleep and Alzheimer disease pathology–a bidirectional relationship. Nat Rev Neurol.

[bib0165] Ju Y.E., Finn M.B., Sutphen C.L., Herries E.M., Jerome G.M., Ladenson J.H., Crimmins D.L., Fagan A.M., Holtzman D.M. (2016). Obstructive sleep apnea decreases central nervous system-derived proteins in the cerebrospinal fluid. Ann. Neurol..

[bib0170] Kang J.E., Lim M.M., Bateman R.J., Lee J.J., Smyth L.P., Cirrito J.R., Fujiki N., Nishino S., Holtzman D.M. (2009). Amyloid-beta dynamics are regulated by orexin and the sleep-wake cycle. Science.

[bib0175] Karran E., Mercken M., De Strooper B. (2011). The amyloid cascade hypothesis for Alzheimer’s disease: an appraisal for the development of therapeutics. Nat. Rev. Drug Discov..

[bib0180] Kerbler G.M., Fripp J., Rowe C.C., Villemagne V.L., Salvado O., Rose S., Coulson E.J., Alzheimer’s Disease Neuroimaging I. (2015). Basal forebrain atrophy correlates with amyloid beta burden in Alzheimer's disease. Neuroimage Clin..

[bib0185] Kylstra W.A., Aaronson J.A., Hofman W.F., Schmand B.A. (2013). Neuropsychological functioning after CPAP treatment in obstructive sleep apnea: a meta-analysis. Sleep Med. Rev..

[bib0190] Lévy P., Kohler M., McNicholas W.T., Barbé F., McEvoy R.D., Somers V.K., Lavie L., Pépin J.-L. (2015). Obstructive sleep apnoea syndrome. Nat. Rev. Dis. Primers.

[bib0195] Lavie L. (2015). Oxidative stress in obstructive sleep apnea and intermittent hypoxia–revisited–the bad ugly and good: implications to the heart and brain. Sleep Med. Rev..

[bib0200] Liguori C., Romigi A., Nuccetelli M., Zannino S., Sancesario G., Martorana A., Albanese M., Mercuri N.B., Izzi F., Bernardini S., Nitti A., Sancesario G.M., Sica F., Marciani M.G., Placidi F. (2014). Orexinergic system dysregulation, sleep impairment, and cognitive decline in Alzheimer disease. JAMA Neurol.

[bib0205] Liguori C., Chiaravalloti A., Izzi F., Nuccetelli M., Bernardini S., Schillaci O., Mercuri N.B., Placidi F. (2017). Sleep apnoeas may represent a reversible risk factor for amyloid-beta pathology. Brain.

[bib0210] Liguori C., Mercuri N.B., Izzi F., Romigi A., Cordella A., Sancesario G., Placidi F. (2017). Obstructive sleep apnea is associated with early but possibly modifiable Alzheimer’s disease biomarkers changes. Sleep.

[bib0215] Lim A.S., Kowgier M., Yu L., Buchman A.S., Bennett D.A. (2013). Sleep fragmentation and the risk of incident Alzheimer’s disease and cognitive decline in older persons. Sleep.

[bib0220] Maestri M., Carnicelli L., Tognoni G., Di Coscio E., Giorgi F.S., Volpi L., Economou N.T., Ktonas P., Ferri R., Bonuccelli U., Bonanni E. (2015). Non-rapid eye movement sleep instability in mild cognitive impairment: a pilot study. Sleep Med..

[bib0225] Mander B.A., Winer J.R., Jagust W.J., Walker M.P. (2016). Sleep: a novel mechanistic pathway, biomarker, and treatment target in the pathology of alzheimer's disease?. Trends Neurosci..

[bib0230] Mateika J.H., Komnenov D. (2017). Intermittent hypoxia initiated plasticity in humans: a multipronged therapeutic approach to treat sleep apnea and overlapping co-morbidities. Exp. Neurol..

[bib0235] Moran M., Lynch C.A., Walsh C., Coen R., Coakley D., Lawlor B.A. (2005). Sleep disturbance in mild to moderate Alzheimer’s disease. Sleep Med..

[bib0240] Nardone R., Bergmann J., Brigo F., Holler Y., Schwenker K., Florea C., Kunz A.B., Golaszewski S., Trinka E. (2016). Cortical afferent inhibition reflects cognitive impairment in obstructive sleep apnea syndrome: a TMS study. Sleep Med..

[bib0245] Ngo H.V., Martinetz T., Born J., Molle M. (2013). Auditory closed-loop stimulation of the sleep slow oscillation enhances memory. Neuron.

[bib0250] Osorio R.S., Gumb T., Pirraglia E., Varga A.W., Lu S.E., Lim J., Wohlleber M.E., Ducca E.L., Koushyk V., Glodzik L., Mosconi L., Ayappa I., Rapoport D.M., de Leon M.J., Alzheimer’s Disease Neuroimaging I. (2015). Sleep-disordered breathing advances cognitive decline in the elderly. Neurology.

[bib0255] Otte A., Turkheimer F., Rosenzweig I. (2016). All you need is sleep. EBioMedicine.

[bib0260] Pan W., Kastin A.J. (2014). Can sleep apnea cause Alzheimer’s disease?. Neurosci. Biobehav. Rev..

[bib0265] Pase M.P., Himali J.J., Grima N.A., Beiser A.S., Satizabal C.L., Aparicio H.J., Thomas R.J., Gottlieb D.J., Auerbach S.H., Seshadri S. (2017). Sleep architecture and the risk of incident dementia in the community. Neurology.

[bib0270] Peppard P.E., Young T., Barnet J.H., Palta M., Hagen E.W., Hla K.M. (2013). Increased prevalence of sleep-disordered breathing in adults. Am. J. Epidemiol..

[bib0275] Rolls A., Shechter R., London A., Ziv Y., Ronen A., Levy R., Schwartz M. (2007). Toll-like receptors modulate adult hippocampal neurogenesis. Nat. Cell Biol..

[bib0280] Rosenzweig I., Kempton M.J., Crum W.R., Glasser M., Milosevic M., Beniczky S., Corfield D.R., Williams S.C., Morrell M.J. (2013). Hippocampal hypertrophy and sleep apnea: a role for the ischemic preconditioning?. PLoS One.

[bib0285] Rosenzweig I., Williams S.C., Morrell M.J. (2013). Crosstalk opposing view: the intermittent hypoxia attending severe obstructive sleep apnoea does not lead to alterations in brain structure and function. J. Physiol..

[bib0290] Rosenzweig I., Williams S.C., Morrell M.J. (2014). The impact of sleep and hypoxia on the brain: potential mechanisms for the effects of obstructive sleep apnea. Curr. Opin. Pulm. Med..

[bib0295] Rosenzweig I., Glasser M., Polsek D., Leschziner G.D., Williams S.C., Morrell M.J. (2015). Sleep apnoea and the brain: a complex relationship. Lancet Respir. Med..

[bib0300] Rosenzweig I., Glasser M., Crum W.R., Kempton M.J., Milosevic M., McMillan A., Leschziner G.D., Kumari V., Goadsby P., Simonds A.K., Williams S.C., Morrell M.J. (2016). Changes in neurocognitive architecture in patients with obstructive sleep apnea treated with continuous positive airway pressure. EBioMedicine.

[bib0305] Sherpa A.D., van de Nes P., Xiao F., Weedon J., Hrabetova S. (2014). Gliotoxin-induced swelling of astrocytes hinders diffusion in brain extracellular space via formation of dead-space microdomains. Glia.

[bib0310] Sherpa A.D., Xiao F., Joseph N., Aoki C., Hrabetova S. (2016). Activation of beta-adrenergic receptors in rat visual cortex expands astrocytic processes and reduces extracellular space volume. Synapse.

[bib0315] Smith A.J., Yao X., Dix J.A., Jin B.J., Verkman A.S. (2017). Test of the ‘glymphatic' hypothesis demonstrates diffusive and aquaporin-4-independent solute transport in rodent brain parenchyma. Elife.

[bib0320] Tahmasian M., Rosenzweig I., Eickhoff S.B., Sepehry A.A., Laird A.R., Fox P.T., Morrell M.J., Khazaie H., Eickhoff C.R. (2016). Structural and functional neural adaptations in obstructive sleep apnea: an activation likelihood estimation meta-analysis. Neurosci. Biobehav. Rev..

[bib0325] Troussiere A.C., Charley C.M., Salleron J., Richard F., Delbeuck X., Derambure P., Pasquier F., Bombois S. (2014). Treatment of sleep apnoea syndrome decreases cognitive decline in patients with Alzheimer’s disease. J. Neurol. Neurosurg. Psychiatry.

[bib0330] Twigg G.L., Papaioannou I., Jackson M., Ghiassi R., Shaikh Z., Jaye J., Graham K.S., Simonds A.K., Morrell M.J. (2010). Obstructive sleep apnea syndrome is associated with deficits in verbal but not visual memory. Am. J. Respir. Crit. Care Med..

[bib0335] Westerberg C.E., Mander B.A., Florczak S.M., Weintraub S., Mesulam M.M., Zee P.C., Paller K.A. (2012). Concurrent impairments in sleep and memory in amnestic mild cognitive impairment. J. Int. Neuropsychol. Soc..

[bib0340] Xanthos D.N., Sandkuhler J. (2014). Neurogenic neuroinflammation: inflammatory CNS reactions in response to neuronal activity. Nat. Rev. Neurosci..

[bib0345] Xie L., Kang H., Xu Q., Chen M.J., Liao Y., Thiyagarajan M., O’Donnell J., Christensen D.J., Nicholson C., Iliff J.J., Takano T., Deane R., Nedergaard M. (2013). Sleep drives metabolite clearance from the adult brain. Science.

[bib0350] Yaffe K., Laffan A.M., Harrison S.L., Redline S., Spira A.P., Ensrud K.E., Ancoli-Israel S., Stone K.L. (2011). Sleep-disordered breathing, hypoxia, and risk of mild cognitive impairment and dementia in older women. JAMA.

[bib0355] Yaffe K., Falvey C.M., Hoang T. (2014). Connections between sleep and cognition in older adults. Lancet Neurol..

[bib0360] Zhu Y., Fenik P., Zhan G., Mazza E., Kelz M., Aston-Jones G., Veasey S.C. (2007). Selective loss of catecholaminergic wake active neurons in a murine sleep apnea model. J. Neurosci..

